# Cost-effectiveness analysis of pembrolizumab plus chemotherapy versus placebo plus chemotherapy for patients with previously untreated locally recurrent inoperable or metastatic triple-negative breast cancer in China

**DOI:** 10.3389/fphar.2025.1654177

**Published:** 2025-08-22

**Authors:** Yibing Hou, Shuo Yang, Xiaohui Wang, Shan Zhao, Huanlong Liu, Shuo Kang

**Affiliations:** ^1^ Department of Pharmacy, The Second Hospital of Hebei Medical University, Shijiazhuang, Hebei, China; ^2^ School of Pharmacy, Hebei Medical University, Shijiazhuang, Hebei, China; ^3^ Department of Oncology, The Second Hospital of Hebei Medical University, Shijiazhuang, Hebei, China; ^4^ Medical Insurance Office, The Second Hospital of Hebei Medical University, Shijiazhuang, Hebei, China

**Keywords:** triple-negative breast cancer, cost-effectiveness, pembrolizumab, placebo, markov model

## Abstract

**Background:**

The present study aimed to evaluate the cost-effectiveness of pembrolizumab combined with chemotherapy versus placebo plus chemotherapy for patients with previously untreated locally recurrent inoperable or metastatic triple-negative breast cancer from the perspective of the Chinese healthcare system.

**Methods:**

A Markov model was developed to track patients’ transitions over 3-week cycles and evaluate the health and economic outcomes over a 10-year horizon for the two competing treatments. The survival data were gathered from the KEYNOTE-355 trial, and cost and utility values were obtained from the published studies. Total costs, life-years, quality-adjusted life-years (QALYs), and incremental cost-effectiveness ratio (ICER) were the model outcomes. We conducted analysis based on patients’ programmed death-ligand 1 (PD-L1) combined positive score (CPS), including subgroups with CPS≥10, CPS≥1, and the intention-to-treat population. One-way sensitivity analysis, probabilistic sensitivity analysis and scenario analysis were performed to examine the robustness of the model results.

**Results:**

In the base case analysis for patients highly expressing PD-L1 (CPS≥10), pembrolizumab plus chemotherapy yielded a marginal cost of $85,838.75 and an additional 0.47 QALYs, resulting in an ICER of $184,030.56 per additional QALY gained, which exceeded the willingness-to-pay (WTP) threshold of $38,224 per QALY in China. And the ICERs were $319,506.90/QALY for patients lowly expressing PD-L1 (CPS≥1) and $776,786.75/QALY for the intention-to-treat population. Sensitivity analyses confirmed the robustness of the model outcomes. Scenario analysis demonstrated that price reductions for pembrolizumab could enhance its likelihood of achieving cost-effectiveness.

**Conclusion:**

The findings of this cost-effectiveness analysis suggest that pembrolizumab plus chemotherapy was not a cost-effective treatment for patients with previously untreated locally recurrent inoperable or metastatic triple-negative breast cancer in China.

## 1 Introduction

Breast cancer is the most prevalent malignancy among women globally, with 2,308,897 new cases worldwide in 2022, making it the leading cause of cancer-related deaths among women (Bray et al., 2024). Triple-negative breast cancer (TNBC), a clinically aggressive subtype, is characterized by the absence of key receptors—specifically estrogen receptor (ER), progesterone receptor (PR), and human epidermal growth factor receptor 2 (HER2) (Patel et al., 2024). TNBC is notorious for its aggressive clinical behavior, characterized by high invasiveness, a pronounced propensity for early metastasis, elevated recurrence rates following treatment, and ultimately, a dismal prognosis with limited therapeutic options (Cho et al., 2021). Due to its poor prognosis and lack of actionable targets for targeted therapies, chemotherapy has long been the primary treatment option for TNBC (Ye et al., 2023). However, its limited efficacy and significant toxicity profile underscore the critical unmet need for novel therapeutic strategies. In recent years, immunotherapy has made great progress in patients with TNBC, particularly for PD-1-positive patients. Immune checkpoint inhibitors (ICIs) have brought more survival benefits to patients, which would enhance the anti-tumor immune function of T cells by inhibiting the immune checkpoint and ligand binding on the surface of patients’ immune cells, thus playing a role in killing tumor cells (Zhang et al., 2021). It is a novel treatment model for patients with previously untreated locally recurrent inoperable or metastatic TNBC. Pembrolizumab is a humanized IgG4 monoclonal antibody with high specificity of binding to the programmed cell death-protein 1 (PD-1), effectively blocking its immunosuppressive signaling pathway and enabling enhanced antitumor immune responses (Kwok et al., 2016).

Recently, Javier Cortes et al. conducted a randomized, double-blind phase three trial, KEYNOTE-355, to assess the efficacy and safety of pembrolizumab combined with chemotherapy in comparison to placebo alongside chemotherapy for patients diagnosed with previously untreated locally recurrent inoperable or metastatic TNBC (Cortes et al., 2020). Compared to the group receiving placebo and chemotherapy, participants receiving pembrolizumab plus chemotherapy represented improvements in terms of overall survival (OS) and progression-free survival (PFS). For patients with combined positive score (CPS)≥10, pembrolizumab plus chemotherapy could significantly improve median OS (23.0 months vs. 16.1 months, hazard ratio [HR] = 0.73, 95% confidence interval [CI]: 0.55–0.95]) and median PFS (9.7 months vs. 5.6 months, HR = 0.65, 95% CI: 0.49–0.86]) in comparison with placebo and chemotherapy. For patients with CPS≥1 and the intention-to-treat population, the median PFS was 7.6 months vs. 5.6 months (HR = 0.74, 95% CI: 0.61–0.90) and 7.5 months vs. 5.6 months (HR = 0.82, 95% CI: 0.69–0.97), respectively. Despite this, no substantial enhancement in OS was observed. Meanwhile, pembrolizumab plus chemotherapy exhibited a manageable safety profile in patients with TNBC. However, there is a lack of comprehensive evaluation regarding the cost-effectiveness of pembrolizumab in combination with chemotherapy across the three subgroups. Cost-effectiveness analysis will not only offer valuable insights for clinicians and policymakers but also play an important role in the decision to reimburse healthcare providers for pharmaceuticals worldwide. The objective of our current study was to assess the cost-effectiveness of pembrolizumab plus chemotherapy compared with placebo plus chemotherapy for patients with previously untreated locally recurrent inoperable or metastatic TNBC from the perspective of Chinese healthcare system.

## 2 Methods

### 2.1 Analytical overview and model structure

**FIGURE 1:**
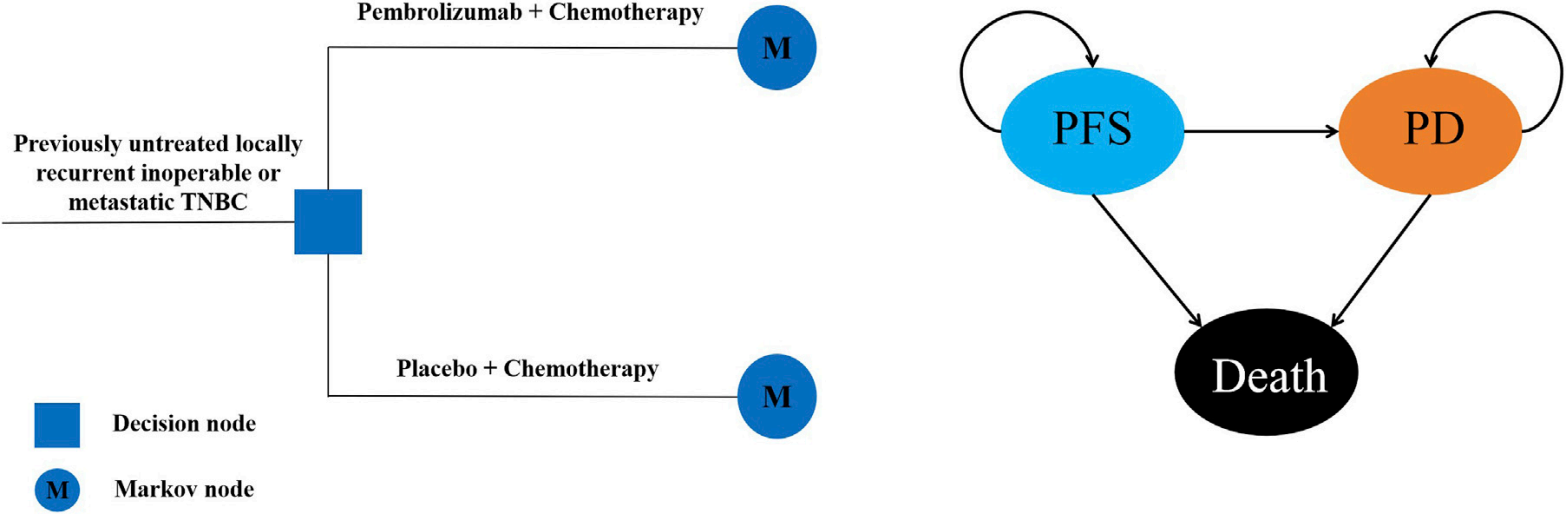
Structure of Markov model PFS, progression-free survival; PD, progressed disease.

A Markov model including three mutually exclusive health states named progression-free survival (PFS), progressed disease (PD) and death was established to analyze the costs and clinical outcomes. In the model, patients received two competing first-line treatments: pembrolizumab plus chemotherapy or placebo plus chemotherapy once every 3 weeks. And the time horizon of the model was set to 10 years, in order to capture most future outcomes and costs associated with the treatment strategies. All patients obtained from the KEYNOTE-355 trial were at the PFS state when entered the model, as the disease continued to progress, patients may remain in the PFS state or enter the state of PD or Death (Figure 1). The primary outcomes measured were total costs, quality-adjusted life-years (QALYs) and the incremental cost-effectiveness ratio (ICER). An annual discount rate of 5% was used to account for the costs and benefits (Liu et al., 2020). All the costs were converted to 2024 US dollars (US $1 = CNY 7.309). The willingness-to-pay (WTP) threshold was set as three times of *per capita* gross domestic product (GDP) of China in 2023 ($38,224/QALY) based on the WHO recommendations (Thokala et al., 2018; Eichler et al., 2004).

### 2.2 Clinical data and transition probabilities

The survival data used in the model were obtained from the KEYNOTE-355 trial, which specifically enrolled patients with previously untreated locally recurrent inoperable or metastatic triple-negative breast cancer, identical to our target population. The trial stratified patients by programmed death-ligand 1 (PD-L1) CPS, with efficacy analysis conducted in three populations: the CPS≥10 subgroup, the CPS≥1 subgroup (which includes all CPS≥10 patients) and the intention-to-treat population. Long-term survival data were obtained by fitting parametric distributions and extrapolating the PFS curve and OS curves from this clinical trial. In the Markov model, time-dependent transition probabilities between states were then calculated using the parameters derived from these survival curves.

We gathered the time and survival rate from the original K-M curves by using the GetData Graph Digitizer V2.26 software. After that, we reconstructed the individual patient data (IPD) and conducted parametric distribution fitting for the survival curves by using the R language 4.4.2 software. The parametric survival model includes exponential, Gamma, Weibull, Log-normal, Log-logistic, Gompertz and Royston/Parmar spline model (Andersson et al., 2011; Royston and Parmar, 2002). To address uncertainty in model selection, we employed a systematic approach. The optimal model was selected based on the Akaike information criterion (AIC), Bayesian Information Criterion (BIC), and visual inspection between the fitting model and K-M curves (Williams et al., 2017). The smaller the AIC and BIC values, the better of the model fitting (Ishak et al., 2013). The AIC and BIC values of the all parametric survival models were shown in Supplementary Table S1. The distribution parameters of the each curve were shown in Table 1. Log-normal survival function S(t) = 1−Φ[(lnt−μ)/σ], log-logistic survival function S(t) = 1/(1+λt^γ^) and generalized gamma survival function were used to calculate the time-dependency transition probabilities (Djalalov et al., 2019).

**TABLE 1:** Survival model parameters fitting to the PFS and OS data from the KEYNOTE-355 trial.

Patients with CPS≥10	PFS		OS	
	Model	Parameters	Model	Parameters
Pembrolizumab plus chemotherapy	Log-normal	Meanlog = 2.3487 Sdlog = 1.2258	Log-normal	Meanlog = 3.1454 Sdlog = 1.1058
Placebo plus chemotherapy	Log-logistic	Shape = 1.763 Scale = 6.618	Log-logistic	Shape = 1.702 Scale = 16.631

Patients with CPS≥1	PFS		OS	
	Model	Parameters	Model	Parameters
Pembrolizumab plus chemotherapy	Generalized gamma	Mu = 1.8691 Sigma = 1.1493	Log-logistic	Shape = 1.641 Scale = 17.819
Placebo plus chemotherapy	Log-logistic	Shape = 1.922 Scale = 6.190	Log-logistic	Shape = 1.902 Scale = 16.164

Intention-to-treat patients	PFS		OS	
	Model	Parameters	Model	Parameters
Pembrolizumab plus chemotherapy	Generalized gamma	Mu = 1.8910 Sigma = 1.1408	Log-logistic	Shape = 1.7151 Scale = 17.2814
Placebo plus chemotherapy	Log-logistic	Shape = 1.816 Scale = 6.068	Log-logistic	Shape = 1.870 Scale = 15.734

PFS: progression-free survival, OS: overall survival.

CPS≥1: PD-L1 CPS, of 1 or higher, CPS≥10: PD-L1 CPS, of 10 or higher.

We assumed that the transition probability of PFS state to Death (pFTD) was equal to the mortality rate of the Chinese general population. The probability of remaining in the PFS state in the next cycle (pFTF) was calculated using the formula: pFTF = S(t)/S (t-1), where S(t) represents the survival probability at time t. Consequently, the transition probability from PFS to PD, denoted as pFTP, was derived as: pFTP = 1- pFTD - pFTF. Similarly, the transition probability from overall survival (including patients in both PFS and PD states) to survival (pSTS) was estimated. Based on this, the transition probability from PD to PD (pPTP) was calculated using the following formula: pPTP = [(nPFS + nPD)*pSTS - (nPFS*pFTF) - (nPFS*pFTP)]/nPD, where nPFS and nPD represent the number of patients in the PFS and PD states, respectively, during the previous Markov cycle. The transition probability from PD to death (pPTD) was then determined as: pPTD = 1 - pPTP.

### 2.3 Cost and utility value

The study was conducted from the perspective of the Chinese healthcare system. Therefore, only direct medical costs were involved, including the drug costs of the pembrolizumab and first-line chemotherapy, supportive treatment, routine follow-up, palliative treatment, and management of treatment-related serious adverse events (SAEs, grade≥3). Grade ≥3 adverse event (AE) incidence rates were extracted directly from the KEYNOTE-355 trial protocol. In our study, it was assumed that the body surface area of patients was 1.72 m^2^, the body weight was 65 kg, and the height was 1.64 m, which were used to calculate the drug dosages and estimate drug costs (Zhu et al., 2018). The prices of therapeutic drugs were obtained from the YAOZHI database as the average of the bid prices for drug procurement around the country (Yaozhi, 2025). The other relevant cost data were from published literature, and costs were adjusted according to the consumer price index (CPI) (National Bureau of Statistics, 2025). Health-state utilities, measured on a scale from 0 (death) to 1 (perfect health), were assigned to approximate QALYs. The utility values of health states were derived from published literature, and the utility values of PFS, PD, and Death were 0.76, 0.55, and 0, respectively (Cai et al., 2024). The specific parameters and their distributions are shown in Table 2.

**TABLE 2:** Model inputs: baseline values and ranges for sensitivity analyses.

Parameter	Range				
	Baseline value	Lower bound	Upper bound	Distribution	References
Cost values (US $)					
Pembrolizumab per 100 mg	2,451.60	1,838.70	3,064.50	Gamma	Yaozhi (2025)
Gemcitabine per 100 mg	18.64	13.98	23.30	Gamma	Yaozhi (2025)
Carboplatin per 100 mg	7.06	5.29	8.82	Gamma	Yaozhi (2025)
Nab-paclitaxel per 100 mg	20.25	15.19	25.31	Gamma	Yaozhi (2025)
Paclitaxel per 100 mg	24.75	18.56	30.94	Gamma	Yaozhi (2025)
Anemia per event	607.06	455.3	758.83	Gamma	Zheng et al. (2024)
Neutropenia per event	547.5	410.63	684.38	Gamma	Zheng et al. (2024)
Neutrophil count decreased per event	104.95	78.71	131.19	Gamma	Zhu et al. (2023)
Supportive treatment per cycle	359	269.25	448.75	Gamma	Zheng et al. (2024)
Routine follow-up per cycle	170	127.5	212.5	Gamma	Zhu et al. (2022)
Terminal care	2,325.75	1,744.34	2,907.24	Gamma	Liao and Kang (2024)
Utility values					
PFS	0.76	0.57	0.95	Beta	Cai et al. (2024)
PD	0.55	0.41	0.69	Beta	Cai et al. (2024)
Disutility of toxicities					
Anemia	−0.029	−0.022	−0.036	Beta	Liu et al. (2021)
Neutropenia	−0.012	−0.050	−0.083	Beta	Mistry et al. (2018)
Neutrophil count decreased	0.2	0.149	0.498	Beta	Gong et al. (2022)
Risk of SAEs in pembrolizumab plus chemotherapy group					
Anemia	0.165	0.134	0.196	Beta	Cortes et al. (2020)
Neutropenia	0.297	0.259	0.335	Beta	Cortes et al. (2020)
Neutrophil count decreased	0.174	0.142	0.206	Beta	Cortes et al. (2020)
Risk of SAEs in placebo plus chemotherapy group					
Anemia	0.146	0.104	0.188	Beta	Cortes et al. (2020)
Neutropenia	0.299	0.245	0.353	Beta	Cortes et al. (2020)
Neutrophil count decreased	0.203	0.156	0.251	Beta	Cortes et al. (2020)
Others					
Discount rate	0.05	0	0.08	Beta	Liu et al. (2020)
Body area surface/m^2^	1.72	1.29	2.15	Beta	Yue et al. (2021)

PFS: progression-free survival, PD: progressed disease.

### 2.4 Sensitivity analyses and scenario analysis

One-way sensitivity analyses and probabilistic sensitivity analyses (PSA) were conducted to examine the robustness of model results when parameters changed. In the one-way sensitivity analysis, the impact of a single variable change on the ICER value was calculated one by one according to the upper and lower limit ranges of the variables. The reasonable range for each parameter was determined based on the 95% confidence intervals derived from published studies or by assuming ±25% of the base-case values when the relevant data were not available, and the results of one-way sensitivity analysis were shown in the Tornado diagram (Zhang et al., 2012). To further assess uncertainty, we conducted PSA using Monte Carlo simulations with 1,000 replications. Key model parameters were jointly sampled from the pre-specified statistical distribution: Gamma distributions for cost data, and Beta distributions for utility values and incidence rates. This approach allowed us to evaluate the robustness of extrapolated survival outcomes, with results visualized in cost-effectiveness scatter plots and acceptability curves. Recently, the average price reduction for drugs included in the National Reimbursement Drug List (NRDL) demonstrated a significant upward trend, especially the price discount of some PD-1/PD-L1 inhibitors. We systematically assessed the impact of varying pembrolizumab price discount scenarios on ICERs to provide valuable information for policymakers.

## 3 Results

### 3.1 Base case analysis

From the Chinese healthcare system perspective, in 10-year time horizon, for patients with previously untreated locally recurrent inoperable or metastatic TNBC, pembrolizumab plus chemotherapy could bring more clinical benefit and cost compared with placebo plus chemotherapy. For patients with CPS≥10, pembrolizumab plus chemotherapy yielded a marginal cost of $85,838.75 and an additional 0.47 QALYs, resulting in an ICER of $184,030.56 per additional QALY gained, which was higher than the WTP threshold of $38,224/QALY in China. For patients with CPS≥1, pembrolizumab plus chemotherapy could bring 0.24 QALYs with the incremental cost of $76,234, resulting in the ICER was $319,506.90/QALY. And for the intention-to-treat population, pembrolizumab plus chemotherapy could bring 0.20 QALYs with the incremental cost of $155,263, resulting in the ICER was $776,786.75/QALY. The ICERs obtained by the three subgroups all exceeded the WTP threshold of $38,224/QALY in China, and the base-case results were shown in Table 3. The base-case results revealed that adding pembrolizumab to chemotherapy could not be considered the cost-effective option in China.

**TABLE 3:** The results of base-case analyses.

Subgroup	Treatment	Incremental cost ($)	Incremental QALY	ICER($/QALY)
Patients with CPS≥10	Pembrolizumab group	85,838.75	0.47	184,030.56
	Placebo group	NA	NA	NA
Patients with CPS≥1	Pembrolizumab group	76,234	0.24	319,506.90
	Placebo group	NA	NA	NA
Intention-to-treat patients	Pembrolizumab group	155,263	0.20	776,786.75
	Placebo group	NA	NA	NA

QALYs: quality-adjusted life-years, ICER: incremental cost-effectiveness ratio, CPS≥1: PD-L1 CPS, of 1 or higher, CPS≥10: PD-L1 CPS, of 10 or higher, NA: not applicable.

### 3.2 Sensitivity analysis

**FIGURE 2:**
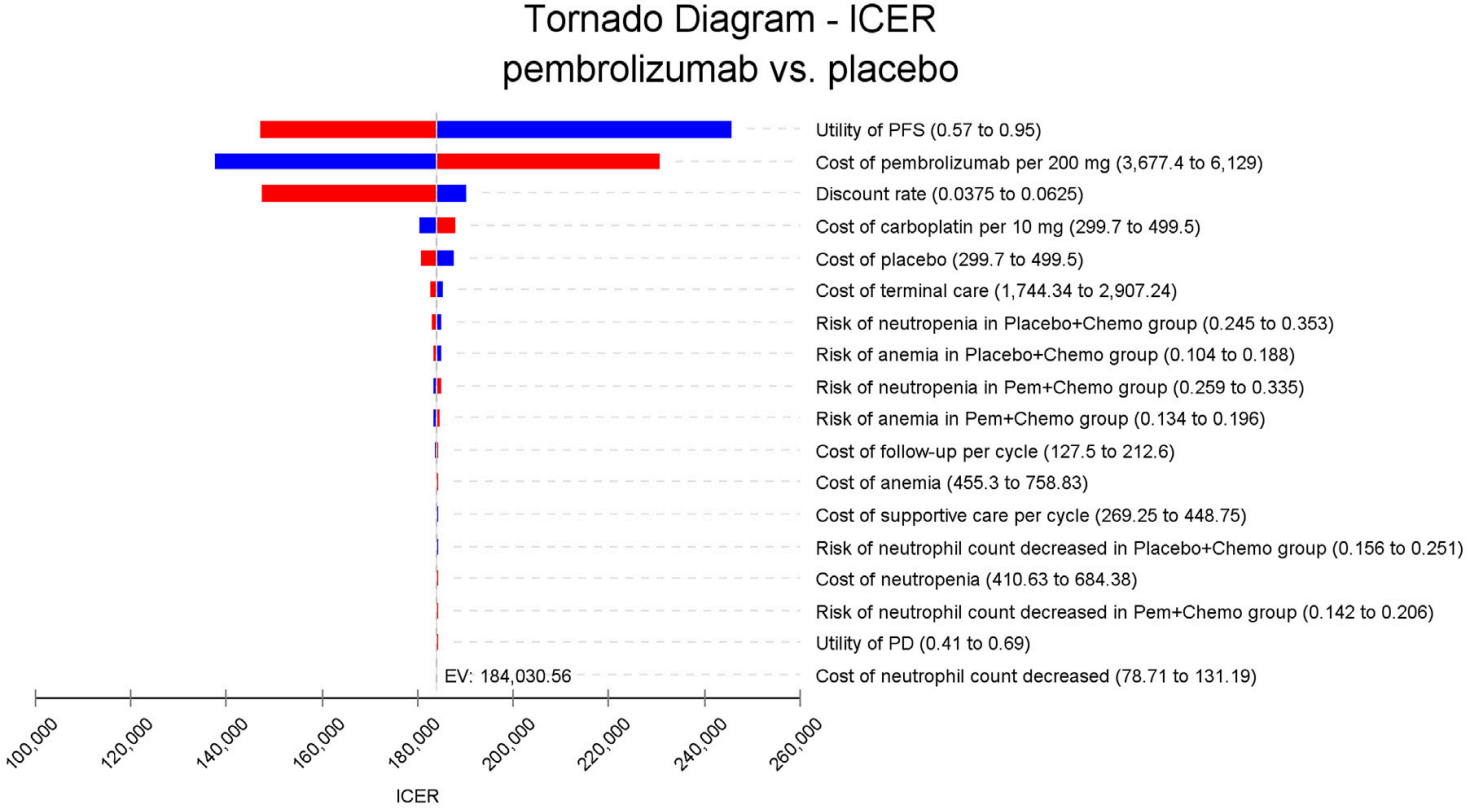
Tornado diagram of one-way sensitivity analyses for patients with CPS≥10. PFS, progression-free survival; PD, progressed disease; ICER, incremental cost-effectiveness ratio; QALY, quality-adjusted life-year; CPS≥1, PD-L1 CPS of 1 or higher; CPS≥10, PD-L1 CPS of 10 or higher.

**FIGURE 3:**
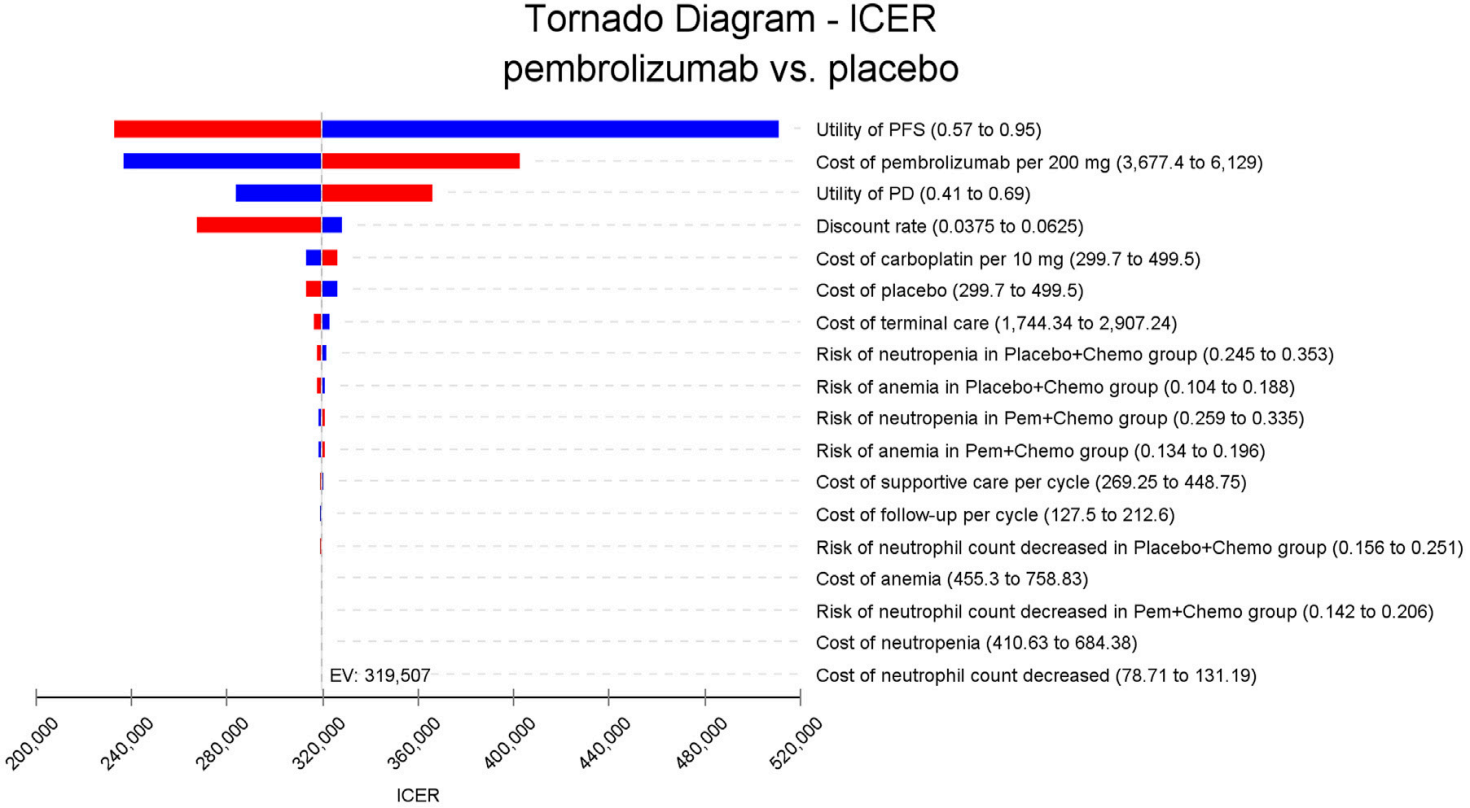
Tornado diagram of one-way sensitivity analyses for patients with CPS≥1.

**FIGURE 4:**
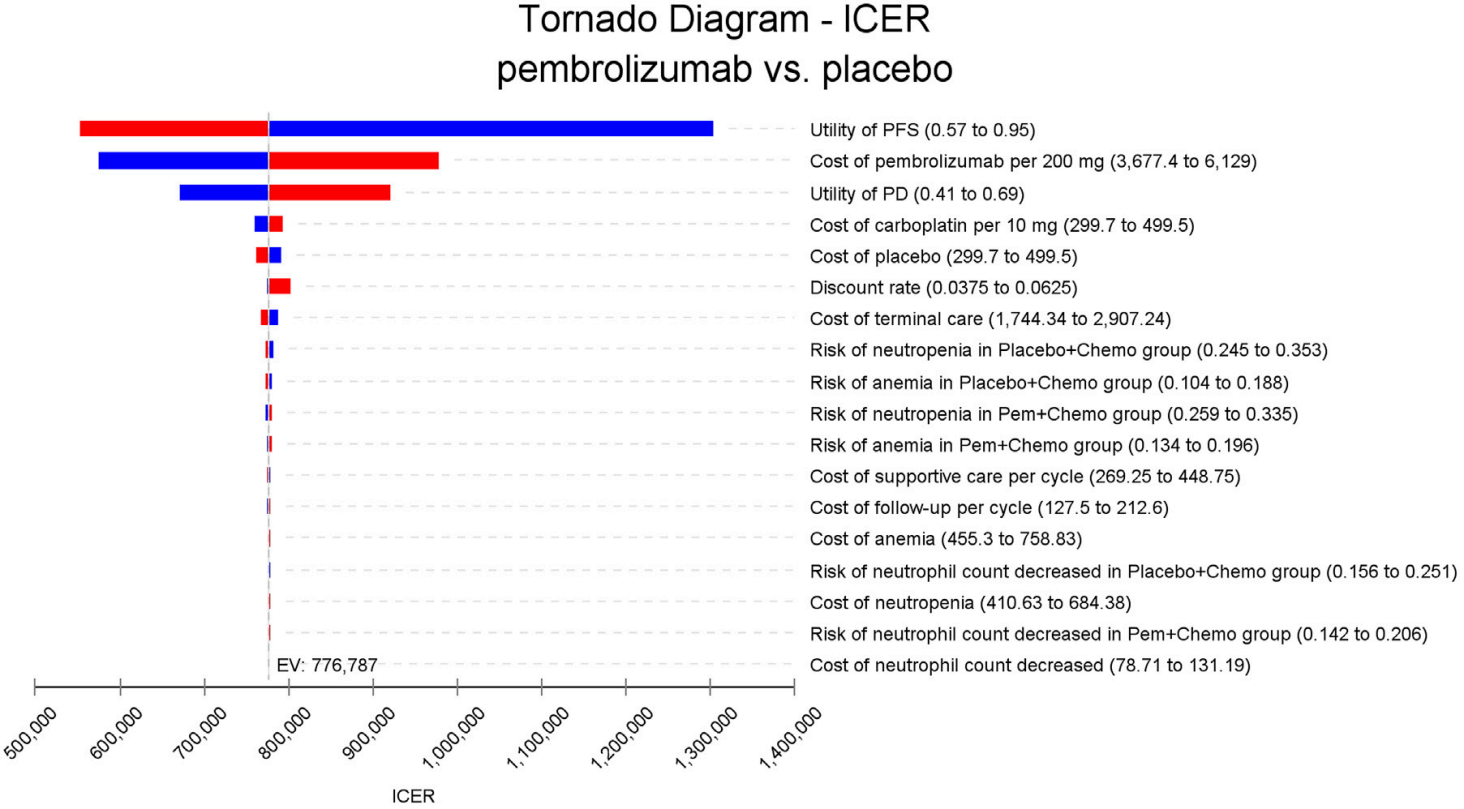
Tornado diagram of one-way sensitivity analyses for the intention-to-treat population.

**FIGURE 5:**
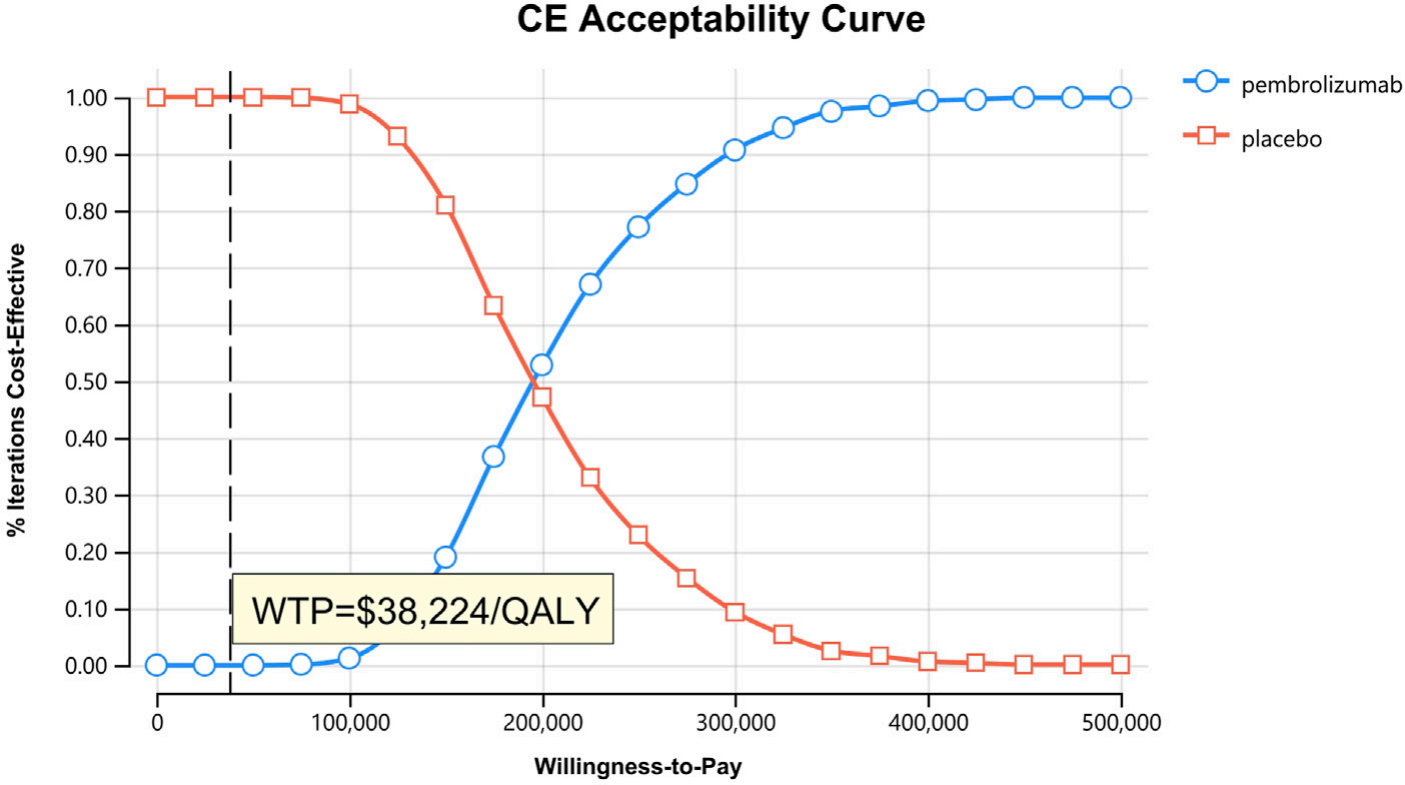
Cost-effectiveness acceptability curves for patients with CPS≥10.

**FIGURE 6:**
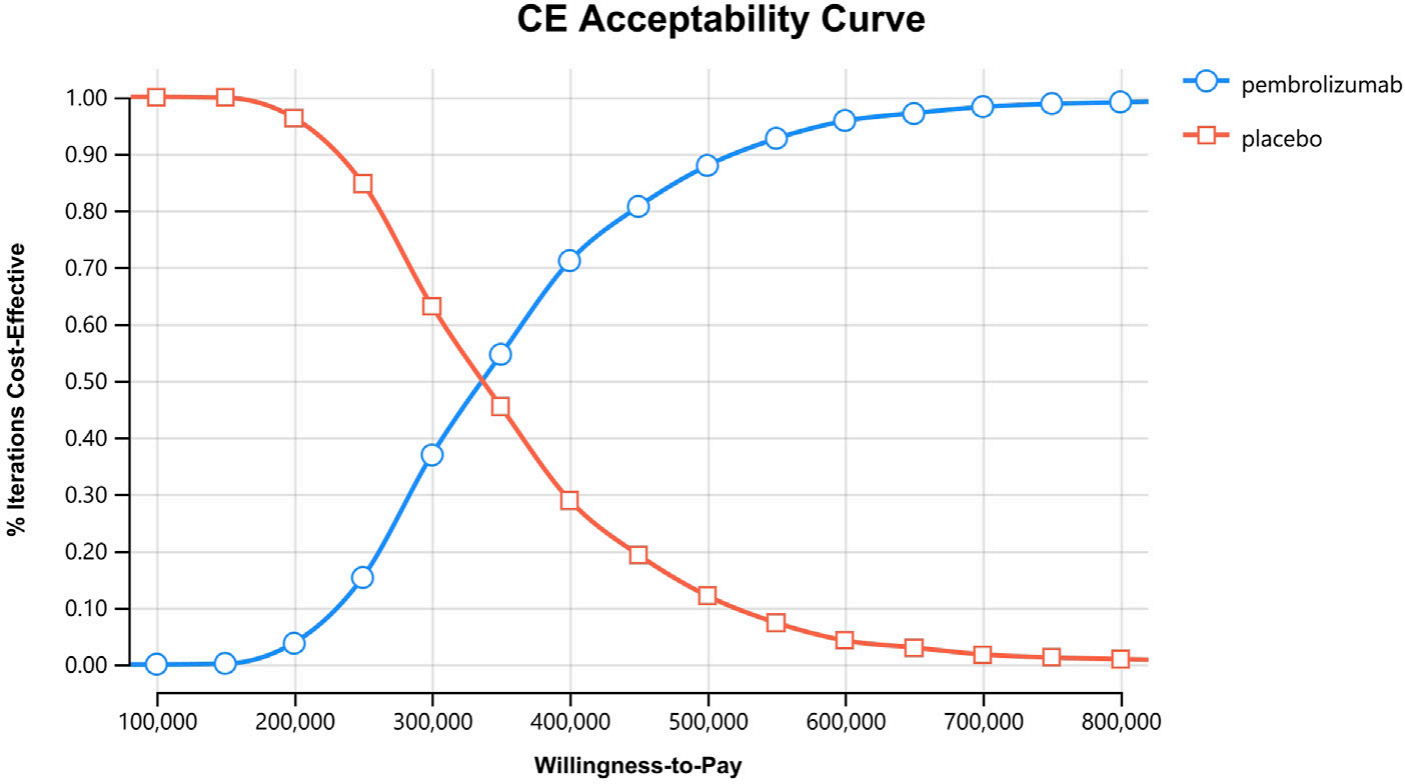
Cost-effectiveness acceptability curves for patients with CPS≥1.

**FIGURE 7:**
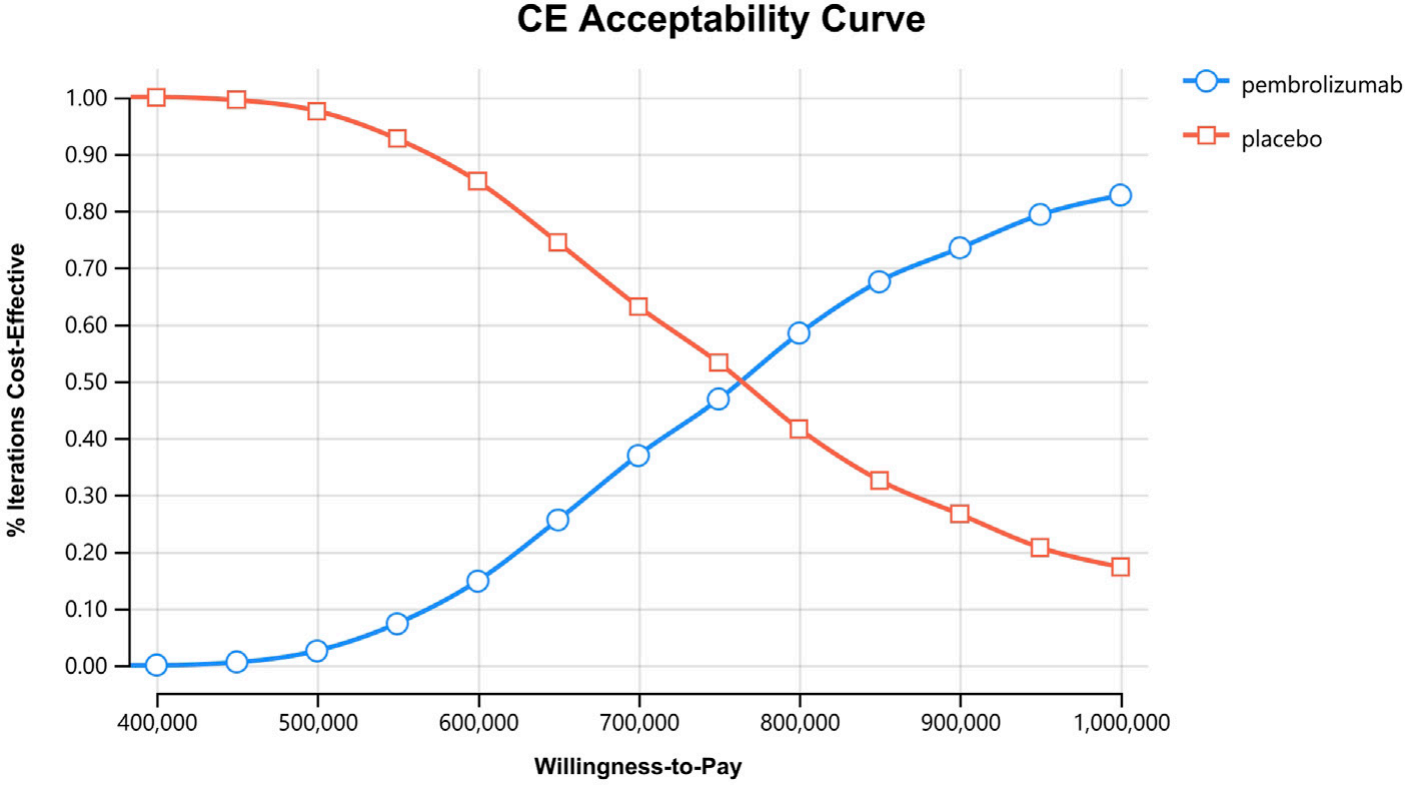
Cost-effectiveness acceptability curves for the intention-to-treat population.

**FIGURE 8:**
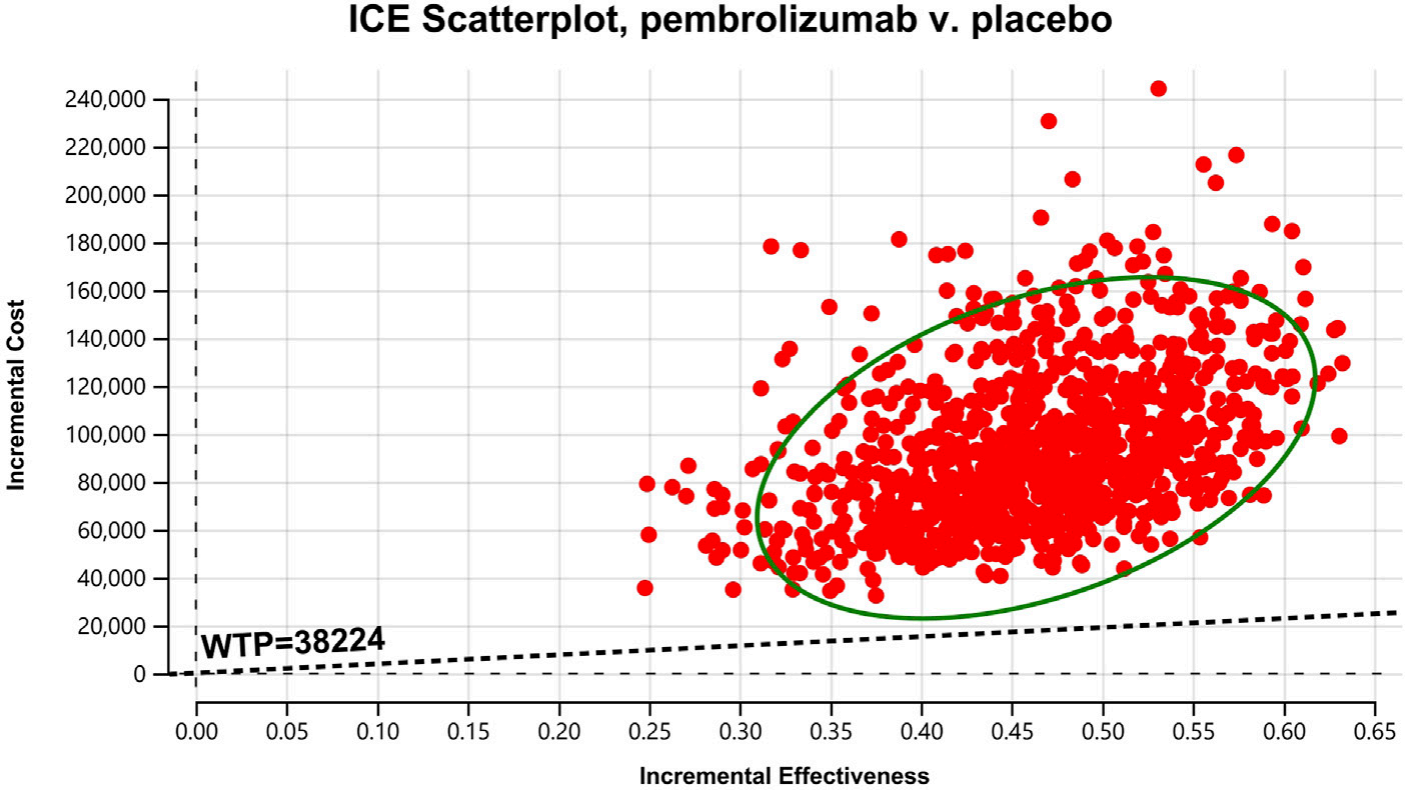
Cost-effectiveness scatter Chart for patients with CPS≥10.

**FIGURE 9:**
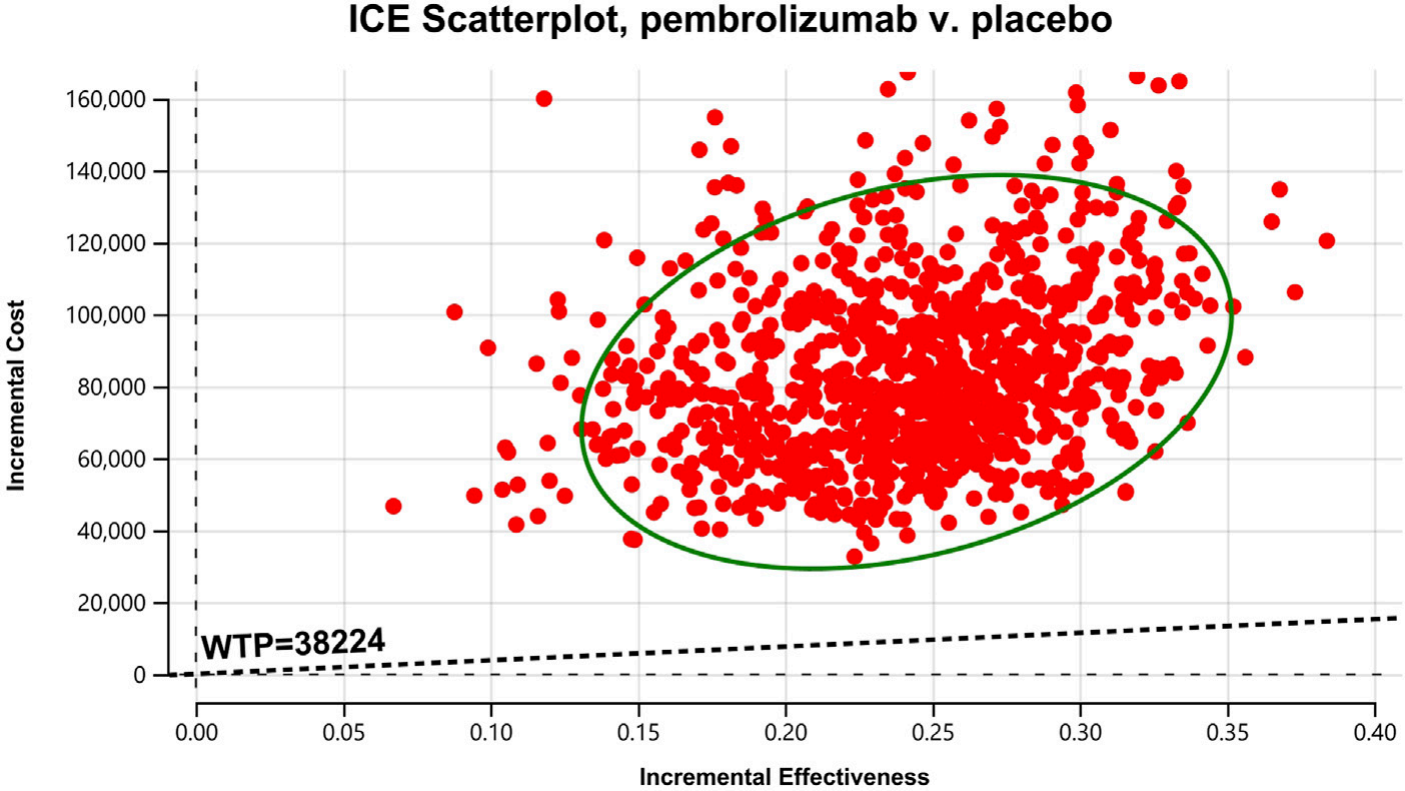
Cost-effectiveness scatter Chart for patients with CPS≥1.

**FIGURE 10:**
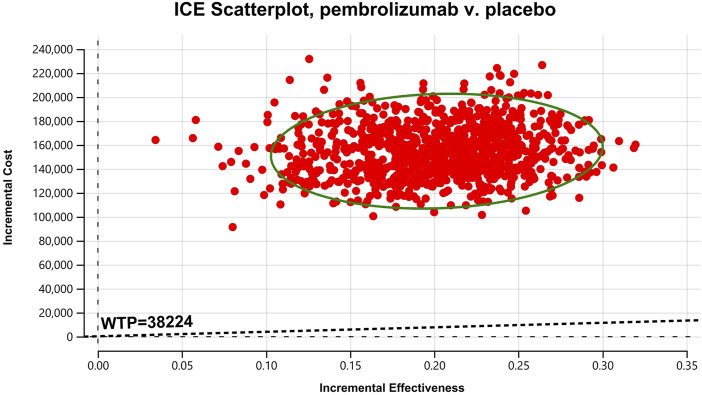
Cost-effectiveness scatter Chart for the intention-to-treat population.

In the one-way sensitivity analyses, results were visualized in a tornado diagram, which highlighted that PFS utility values, PD utility values and the cost of pembrolizumab exerted the greatest influence on the ICER. Parameter variations demonstrated the robustness of model outcomes (Figures 2–4). Probabilistic sensitivity analysis showed a 0% probability that pembrolizumab plus chemotherapy was cost-effective across all three subgroups at China’s WTP threshold of $38,224/QALY, confirming the model’s robustness (Figures 5–10). In the CPS≥10 subgroup, the probability of pembrolizumab being cost-effective increased to 55.4% when the WTP threshold was $200,000/QALY. In the CPS≥1 subgroup, at a WTP threshold of $360,000/QALY, this probability reached 58.0%. And the probability increased to 50.2% when the WTP threshold was $750,000/QALY for the intention-to-treat population. As the WTP threshold increased, the probability of pembrolizumab being economically rose accordingly.

### 3.3 Scenario analysis

We performed scenario analyses evaluating different price reductions for pembrolizumab (80%, 90%, and 95% discount) within the economic models of three subgroups. Our findings demonstrated that as the discount rate of pembrolizumab increased, the ICERs gradually decreased. For patients with CPS≥10, when the cost of pembrolizumab decreased by 80%, the ICER was $35,362/QALY, which was lower than the WTP threshold of $38,224/QALY in China. For patients with CPS≥1 and the intention-to-treat population, the pembrolizumab group was cost-effective compared to the placebo group when the price of pembrolizumab was reduced by 90% and 95%, respectively (Table 4).

**TABLE 4:** The results of scenario analyses.

Subgroup	Treatment	Incremental cost ($)	Incremental QALY	ICER($/QALY)	Discount Rate(%)
Patients with CPS≥10	Pembrolizumab group	16,494.37	0.47	35,362.45	80%
	Placebo group	NA	NA	NA	
Patients with CPS≥1	Pembrolizumab group	4,936	0.24	20,688.76	90%
	Placebo group	NA	NA	NA	
Intention-to-treat patients	Pembrolizumab group	2,011	0.20	10,058.71	95%
	Placebo group	NA	NA	NA	

QALYs: quality-adjusted life-years, ICER: incremental cost-effectiveness ratio, CPS≥1: PD-L1 CPS, of 1 or higher, CPS≥10: PD-L1 CPS, of 10 or higher, NA: not applicable.

## 4 Discussion

Currently, ICIs, either as monotherapy or in combination with other regimens, have emerged as a key therapeutic option for TNBC. In the KEYNOTE-355 trial, pembrolizumab plus chemotherapy statistically extended PFS and OS in patients with previously untreated locally recurrent inoperable or metastatic TNBC compared with placebo plus chemotherapy. However, the financial burden imposed by immuno-oncology drugs for the majority of middle- and low-income patients and the consequent challenges to healthcare system sustainability represent a critical issue in contemporary medical practice. Therefore, it is an urgent agenda to conduct a cost-effectiveness analysis to evaluate their economic impact, particularly in resource-constrained countries like China. In this study, we developed a Markov model to explore the economic ramifications of using pembrolizumab as a primary treatment for Chinese patients with previously untreated locally recurrent inoperable or metastatic TNBC. The results showed that in 10-year horizon, pembrolizumab plus chemotherapy is not cost-effective compared to placebo plus chemotherapy. For patients with CPS≥10, pembrolizumab plus chemotherapy yielded a marginal cost of $85,838.75 and an additional 0.47 QALYs, resulting in an ICER of $184,030.56 per additional QALY gained. For patients with CPS≥1, pembrolizumab plus chemotherapy could bring 0.24 QALYs with the incremental cost of $76,234, resulting in the ICER was $319,506.90 per additional QALY gained. And for the intention-to-treat population, pembrolizumab plus chemotherapy could bring 0.20 QALYs with the incremental cost of $155,263, resulting in the ICER was $776,786.75 per additional QALY gained. The ICERs obtained by the three subgroups all exceeded the WTP threshold of $38,224 in China, indicating that they were not economically viable. Our analysis revealed a clear cost-effectiveness gradient across PD-L1 expression levels. The CPS≥10 subgroup demonstrated better economic outcomes compared to the CPS≥1 subgroup, with higher incremental QALYs and lower ICERs. The one-way sensitivity analysis and probabilistic sensitivity analysis demonstrated that the model outcomes were robustness. The results indicated that adding pembrolizumab to chemotherapy was not a cost-effective treatment for patients with different PD-L1 CPS expression and the intention-to-treat population. Scenario analysis demonstrated that price reductions for pembrolizumab could enhance its likelihood of achieving cost-effectiveness. And differential price reductions were required to achieve cost-effectiveness: an 80% reduction for CPS≥10 patients, 90% for CPS≥1 patients, and 95% for the intention-to-treat population.

Prior clinical trials have consistently demonstrated the therapeutic benefits of immune checkpoint inhibitors as a treatment regimen for TNBC (U.S. National Library of Medicine, 2025; Schmid et al., 2020; Jiang et al., 2024). Up to now, ICIs including pembrolizumab, atezolizumab and toripalimab are available for the treatment for patients with TNBC, and pharmacoeconomic evaluations for TNBC have been increasingly conducted. Based on the KEYNOTE-522 clinical trial, four studies showed that the addition of pembrolizumab in various combinations in patients with TNBC was likely to be cost-effective from the perspectives of different countries (Favre-Bulle et al., 2024; Huang et al., 2023; Kwong et al., 2024; Pöllinger et al., 2025). For the KEYNOTE-355 clinical trial, while two studies supported pembrolizumab’s cost-effectiveness in the US and Japan (Huang et al., 2022; Chisaki et al., 2025), another study questioned its economic value in China (Zhu et al., 2023). Notably, previous studies have not conducted subgroup analyses or evaluated outcome indicators across the three populations: different PD-L1 CPS expression and the intention-to-treat group (Zhu et al., 2023; Huang et al., 2022; Chisaki et al., 2025). Compared with other ICIs, atezolizumab has not shown better affordability in metastatic TNBC (Liu et al., 2021), whereas toripalimab, a domestically developed PD-1 inhibitor in China, may present moderate cost-effectiveness due to its lower price (Cai et al., 2024), though direct comparative studies remain scarce. The translation of clinical benefits into economic value poses particular challenges for China’s healthcare system amid expanding ICI applications, necessitating thorough subgroup economic evaluations to inform precision decision-making.

This study has several limitations that warrant consideration in healthcare decision-making. First, while the clinical data included in the model have been obtained from the KEYNOTE-355 trial, several factors—such as differences in clinical practice patterns, the cost structure of oncology care, patient characteristics could vary across different countries, may limit the generalizability of our findings to the Chinese healthcare setting. Second, utility values were derived from published literature and not locally validated, without distinguishing potential biases among treatment strategies, which may lead to an overestimation of QALY gains. Third, cost data were obtained from published sources rather than real-world settings, future studies should prioritize real-world cost evidence to enhance the generalizability and accuracy of cost estimates. The substantial urban-rural divide in China’s healthcare system merits particular attention, where urban centers typically exhibit 2–3 times higher *per capita* health expenditures compared to rural regions, potentially affecting medication affordability and reimbursement decision applicability across different socioeconomic strata (Tang et al., 2021). Fourth, our study only included the costs of management SAEs (Grade≥3), and ignored the costs of AEs below grade 3, although these cost values only had minimal influence of the model results revealed by one-way sensitivity analyses. Fifth, in this study, we employed a Markov model rather than the partitioned survival model. Although Markov model can better capture the progression of diseases based on their distinct characteristics, they require the calculation of transition probabilities and impose extremely high demands on clinical evidence, making it difficult to ensure accuracy and feasibility. Notably, a literature have demonstrated there was no significant difference in most outcome measures derived from the two models, both of them are recommended to assess model structure uncertainty (Rui et al., 2021). Finally, our evaluation focused solely on pembrolizumab, future studies should incorporate comparative analyses of other ICIs through network meta-analysis approaches. Despite the limitations of the study, the sensitivity analysis demonstrated the robustness of our model outcomes. Therefore, it might provide valuable reference for clinical treatment decisions and policymakers.

Our findings highlight the economic challenges of pembrolizumab adoption in China, with all ICER estimates exceeding the WTP thresholds. These results carry important implications for both clinical practice and health policy. For physicians, our study highlights the need to balance therapeutic benefits with economic burdens when selecting treatment regimens. For policymakers, we emphasizes the importance of considering regional economic variations and alternative ICIs when making reimbursement decisions, particularly in resource-constrained settings. In addition, price negotiation mechanisms should be strengthened for high-cost ICIs, particularly through volume-based procurement approaches that have successfully reduced drug prices in China’s national reimbursement drug list negotiations (Mingge et al., 2023). Future research should focus on comprehensive comparisons of available ICIs and develop region-specific cost-effectiveness thresholds to optimize resource allocation in China’s heterogeneous healthcare landscape.

## 5 Conclusion

In summary, from the Chinese healthcare system perspective, pembrolizumab in combination with chemotherapy has been shown a survival benefit in improving the survival of patients with previously untreated locally recurrent inoperable or metastatic TNBC and has no cost-effectiveness advantages in terms of economics in any subgroups. Price reduction of pembrolizumab may be a potential solution to make it cost-effective.

## Data Availability

The original contributions presented in the study are included in the article/Supplementary Material, further inquiries can be directed to the corresponding authors.
