# Supplementary material for: Cost-effectiveness analysis of pembrolizumab plus chemotherapy versus placebo plus chemotherapy for patients with previously untreated locally recurrent inoperable or metastatic triple-negative breast cancer in China
Source: Front Pharmacol. 2025 Aug 22;16:1654177. doi: 10.3389/fphar.2025.1654177 (PMC12411481; doi:10.3389/fphar.2025.1654177)
Supplement: Supplementary file 1 [file Supplementaryfile1.docx]

Supplementary Figures

**Supplementary Figure S1.** The replicated Kaplan-Meier PFS and OS curves of the two competing regimens with three subgroups in KEYNOTE-355 trial.

Patients with CPS≥10

**
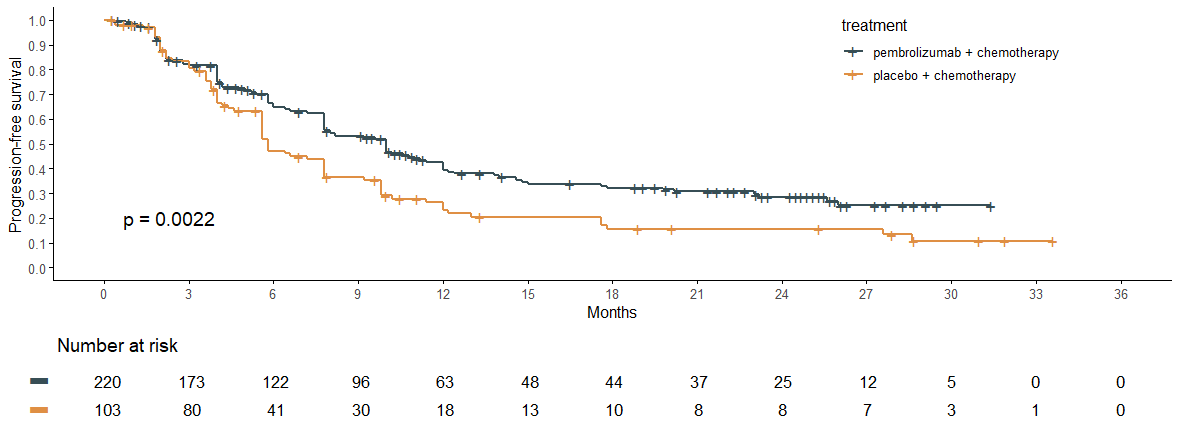
**

**
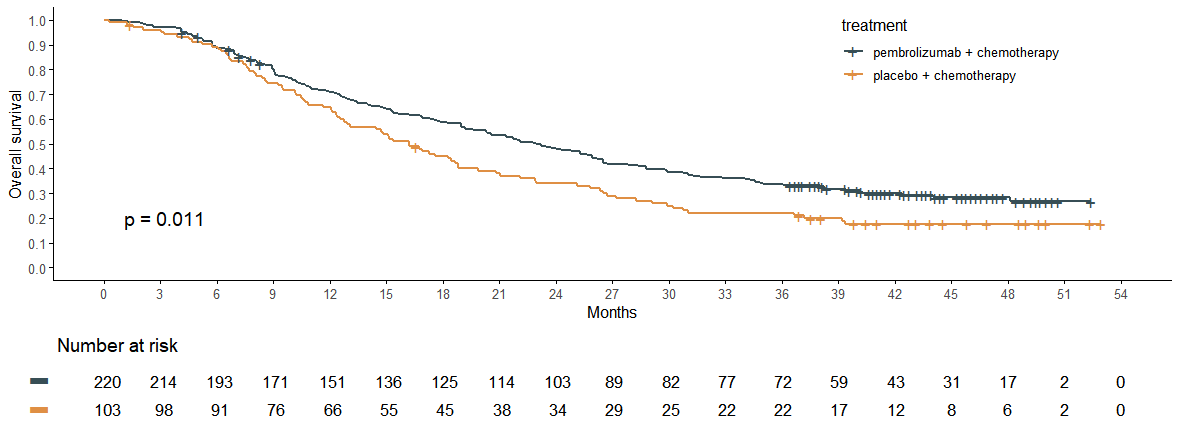
**

Patients with CPS≥1


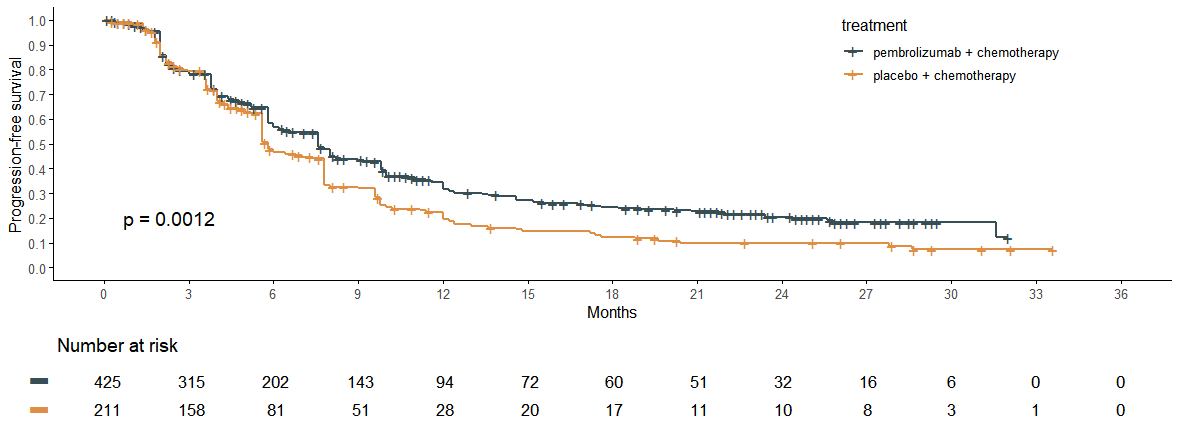


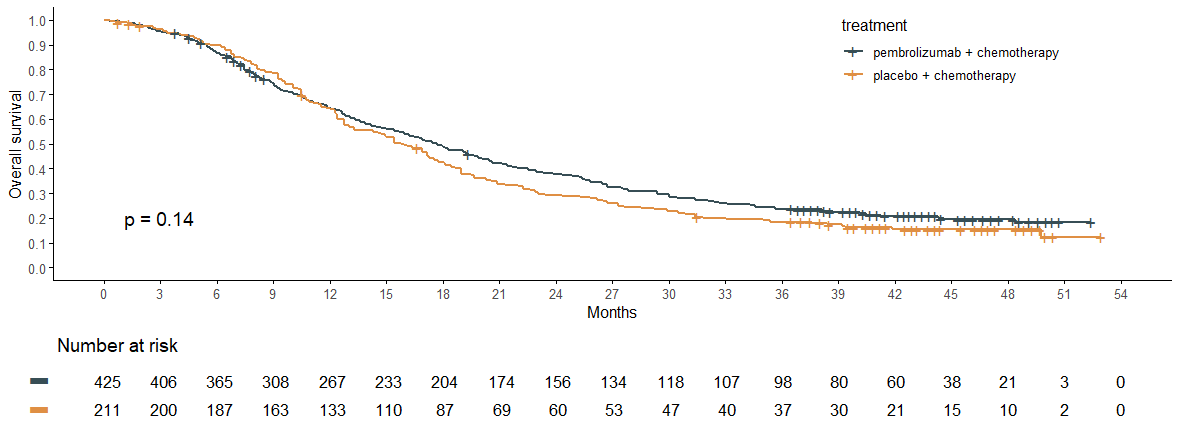


Intention-to-treat patients


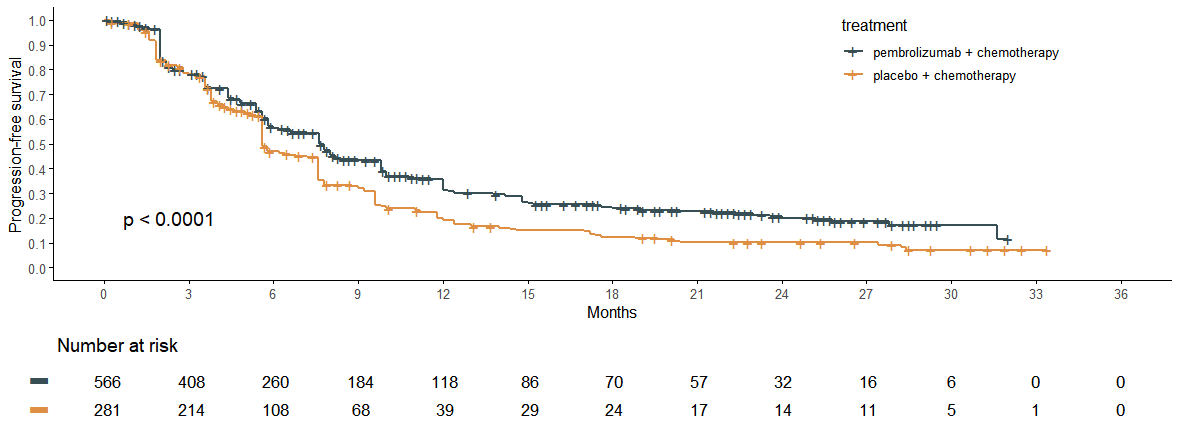


**
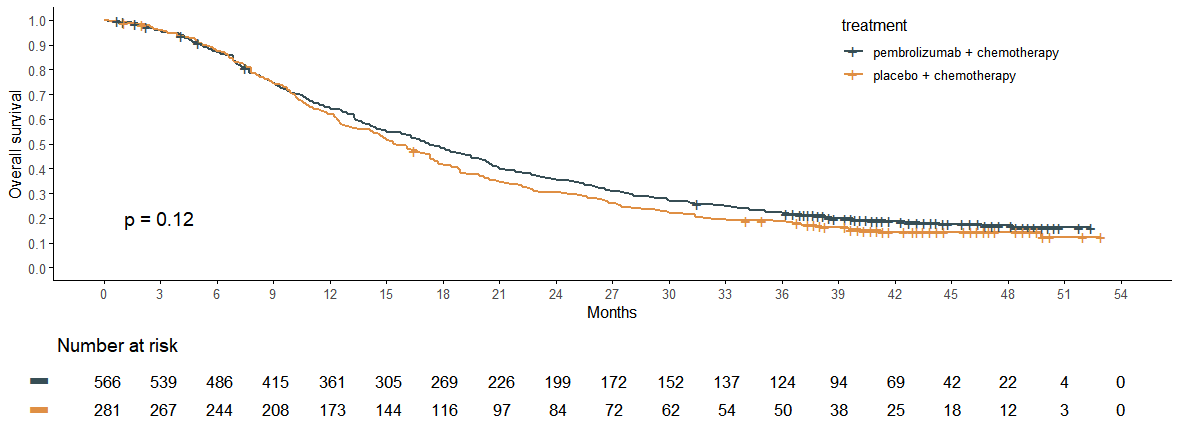
**

**Supplementary Figure S1.** The replicated Kaplan-Meier PFS and OS curves of the two competing regimens with three subgroups in KEYNOTE-355 trial.
