# Supplementary material for: Cost-effectiveness analysis of pembrolizumab plus chemotherapy versus placebo plus chemotherapy for patients with previously untreated locally recurrent inoperable or metastatic triple-negative breast cancer in China
Source: Front Pharmacol. 2025 Aug 22;16:1654177. doi: 10.3389/fphar.2025.1654177 (PMC12411481; doi:10.3389/fphar.2025.1654177)
Supplement: Supplementary file 2 [file Supplementaryfile2.docx]

Supplementary Tables

**Supplementary Table S1** Summary of statistical goodness-of-fit of Kaplan-Meier curves in KEYNOTE-355 trial.

**Supplementary Table S1** Summary of statistical goodness-of-fit of Kaplan-Meier curves in KEYNOTE-355 trial.

| Subgroups | Treatments | Distribution | AIC values of PFS | BIC values of PFS | AIC values of OS | BIC values of OS |
| --- | --- | --- | --- | --- | --- | --- |
| Patients with CPS≥10 | Pembrolizumab plus chemotherapy | Exponential | 1003.3559 | 1006.7496 | 1390.171 | 1393.565 |
|  |  | Gamma | 1004.6749 | 1011.4622 | 1385.543 | 1392.330 |
|  |  | Weibull | 1005.3543 | 1012.1415 | 1388.265 | 1395.052 |
|  |  | Log-normal | 981.4131 | 988.2003 | 1371.809* | 1378.596* |
|  |  | Log-logistic | 987.0373 | 993.8245 | 1374.947 | 1381.735 |
|  |  | Gompertz | 998.4581 | 1005.2454 | 1392.147 | 1398.934 |
|  |  | Generalized gamma | 977.4087* | 987.5896* | 1373.181 | 1383.362 |
|  | Placebo plus chemotherapy | Exponential | 508.1273 | 510.7620 | 702.2065 | 704.8412 |
|  |  | Gamma | 506.6590 | 511.9285 | 699.6943 | 704.9638 |
|  |  | Weibull | 509.0349 | 514.3043 | 701.3984 | 706.6678 |
|  |  | Log-normal | 493.6877 | 498.9572 | 698.6012 | 703.8706 |
|  |  | Log-logistic | 491.7909* | 497.0604* | 692.6562* | 697.9257* |
|  |  | Gompertz | 508.3675 | 513.6369 | 704.2045 | 709.4739 |
|  |  | Generalized gamma | 495.2633 | 503.1675 | 698.4755 | 706.3796 |

| Subgroups | Treatments | Distribution | AIC values of PFS | BIC values of PFS | AIC values of OS | BIC values of OS |
| --- | --- | --- | --- | --- | --- | --- |
| Patients with CPS≥1 | Pembrolizumab plus chemotherapy | Exponential | 1982.731 | 1986.783 | 2848.805 | 2852.857 |
|  |  | Gamma | 1979.307 | 1987.411 | 2834.300 | 2842.404 |
|  |  | Weibull | 1983.557 | 1991.661 | 2840.801 | 2848.905 |
|  |  | Log-normal | 1932.750 | 1940.854* | 2817.135 | 2825.239 |
|  |  | Log-logistic | 1939.649 | 1947.754 | 2809.211* | 2817.315* |
|  |  | Gompertz | 1977.304 | 1985.408 | 2850.804 | 2858.909 |
|  |  | Generalized gamma | 1928.833* | 1940.989 | 2817.407 | 2829.564 |
|  | Placebo plus chemotherapy | Exponential | 1046.671 | 1050.022 | 1448.744 | 1452.095 |
|  |  | Gamma | 1032.799 | 1039.503 | 1431.887 | 1438.590 |
|  |  | Weibull | 1040.507 | 1047.210 | 1437.864 | 1444.568 |
|  |  | Log-normal | 1012.044 | 1018.747 | 1426.581 | 1433.285 |
|  |  | Log-logistic | 1008.048* | 1014.752* | 1414.817* | 1421.520* |
|  |  | Gompertz | 1048.400 | 1055.104 | 1450.001 | 1456.705 |
|  |  | Generalized gamma | 1013.989 | 1024.045 | 1425.906 | 1435.962 |

| Subgroups | Treatments | Distribution | AIC values of PFS | BIC values of PFS | AIC values of OS | BIC values of OS |
| --- | --- | --- | --- | --- | --- | --- |
| Intention-to-treat patients | Pembrolizumab plus chemotherapy | Exponential | 2568.968 | 2573.307 | 3883.098 | 3887.437 |
|  |  | Gamma | 2561.737 | 2570.414 | 3853.910 | 3862.587 |
|  |  | Weibull | 2568.320 | 2576.997 | 3863.965 | 3872.643 |
|  |  | Log-normal | 2502.704 | 2511.381 | 3839.403 | 3848.080 |
|  |  | Log-logistic | 2512.330 | 2521.007 | 3824.647* | 3833.324* |
|  |  | Gompertz | 2563.493 | 2572.171 | 3883.996 | 3892.673 |
|  |  | Generalized gamma | 2498.225* | 2511.240* | 3836.075 | 3849.090 |
|  | Placebo plus chemotherapy | Exponential | 1419.207 | 1422.846 | 1959.697 | 1963.335 |
|  |  | Gamma | 1407.332 | 1414.609 | 1937.708 | 1944.984 |
|  |  | Weibull | 1414.758 | 1422.035 | 1945.273 | 1952.550 |
|  |  | Log-normal | 1385.703 | 1392.980 | 1929.059 | 1936.336 |
|  |  | Log-logistic | 1376.927* | 1384.204* | 1917.681* | 1924.958* |
|  |  | Gompertz | 1419.979 | 1427.256 | 1960.533 | 1967.810 |
|  |  | Generalized gamma | 1387.204 | 1398.119 | 1928.354 | 1939.269 |

* adopted parametric survival function in the model, AIC Akaike information criterion, BIC Bayesian information criterion, PFS progression-free survival, OS overall survival.
